# Associations between pattern separation and hippocampal subfield structure and function vary along the lifespan: A 7 T imaging study

**DOI:** 10.1038/s41598-020-64595-z

**Published:** 2020-05-05

**Authors:** Joost M. Riphagen, Lisa Schmiedek, Ed H. B. M. Gronenschild, Michael A. Yassa, Nikos Priovoulos, Alexander T. Sack, Frans R. J. Verhey, Heidi I. L. Jacobs

**Affiliations:** 1 Faculty of Health, Medicine and Life Sciences, School for Mental Health and Neuroscience, Alzheimer Centre Limburg, Maastricht University, Maastricht, The Netherlands; 2 Department of Neurobiology and Behavior, Center for the Neurobiology of Learning and Memory, University of California, Irvine, CA USA; 3 Faculty of Psychology and Neuroscience, Department of Cognitive Neuroscience, Maastricht University, PO BOX 616, 6200 MD Maastricht, The Netherlands; 4 Gordon Center for Medical Imaging, Department of Radiology, Massachusetts General Hospital and Harvard Medical School, Boston, MA USA

**Keywords:** Cognitive ageing, Hippocampus, Neural ageing

## Abstract

Pattern separation (PS) describes the process by which the brain discriminates similar stimuli from previously encoded stimuli. This fundamental process requires the intact processing by specific subfields in the hippocampus and can be examined using mnemonic discrimination tasks. Previous studies reported different patterns for younger and older individuals between mnemonic discrimination performance and hippocampal subfield activation. Here, we investigated the relationship between the lure discrimination index (LDI) and hippocampal subfield volume and activity across the adult lifespan (20–70 years old). Using ultra-high field functional and structural magnetic resonance imaging at 7 T, we found that lower DG volume and higher CA3 activation was associated with worse LDI performance in individuals (>60 years), suggesting that this higher activation may be an indication of aberrant neurodegenerative-related processes. In fact, higher activation in the CA1 and DG was associated with lower volumes in these subfields. For individuals around 40–50 years old, we observed that greater left and right DG volume, and greater activity in the CA3 was associated with lower LDI performance. Taken together, these results suggest that the relationship between memory and hippocampal subfield structure or function varies nonlinearly and possibly reciprocally with age, with midlife being a critically vulnerable period in life.

## Introduction

A decline in cognitive functions is part of normal ageing^1^ and for older adults, memory problems, in particular in episodic memory, are considered among the most worrisome. The hippocampus is known to play a central role in episodic memory^2,3^. The hippocampus is not a homogeneous structure, different subfields are involved in different memory processes^4^. This has in particular been shown for pattern separation and completion, the most extensively investigated memory processes in the context of distinct hippocampal subfield affinities.

Pattern separation, the ability to form distinct, non-overlapping representations from similar or overlapping inputs has been shown to rely on the dentate gyrus (DG) and Cornu Ammonis (CA3) regions in human studies^5–10^. A distinction is made between behavioural pattern separation of objects and of spatial locations^4^. While the downstream pathways for spatial pattern separation involve medial entorhinal cortices, object pattern separation engages the lateral entorhinal cortices. In the hippocampal subfields CA3/DG the distinction between object and spatial pattern separation is no longer present^11^. Both variants of pattern separation (object and spatial) are similarly affected by age. Age-related changes in pattern separation have been documented extensively in animal^12^ and human studies^4,13^. Atrophy of the DG in rodent models of ageing correlated with discrimination deficits^14^. Behavioural work demonstrated a monotonic decline in discrimination abilities starting in the fourth decade in humans^15^. Positive associations between CA3/DG volume and performance on an object mnemonic discrimination task has been reported in individuals of 60 years of age and older^16^. Longitudinal cohort studies have shown curvilinear relationships between total hippocampal volume and age, with the first changes noticeable around age 40, suggesting that midlife is a critical period in life sensitive for age-related neurodegenerative processes^17,18^.

In addition to structural alterations to hippocampal subfield tissue, mnemonic discrimination deficits were also associated with hyperactivity in DG/CA3 in late adulthood (60–80 years), as measured with functional magnetic resonance imaging (fMRI)^19,20^, that are in contrast to the typically observed flexible modulation of the BOLD response in CA3/DG in younger adults. In the latter, an increase of the activation was observed with a changing similarity of the experience (i.e. greater dissimilarity of the input). This age-related hyperactivation can be reduced using low-dose anti-epileptic drugs^21^ suggesting that it is not a compensatory hyperactivation but rather an index of dysfunction. Evidence from animal studies suggests that this hyperactivation in CA3/DG is linked to loss of inhibitory input from the perforant path to the DG and CA3 regions^12^.

Few studies so far included specifically a middle-aged group when investigating hippocampal activation during an associative recognition memory task^22^. Subtle differences in hippocampal activity between the middle-aged and older groups were found, with the older group having less activation during successful retrieval compared to the middle-aged group. These findings combined with the hyperactivity reports in older individuals suggest that hippocampal activity is non-linearly associated with age. Another study included participants across the entire adult lifespan (range: 19 y–76 y), with a large number of also middle-aged adults (n = 39, age-range: 40–58 y)^23^. This study aimed to investigate age-invariant patterns of brain activation during encoding and retrieval of a memory task and found that in bilateral hippocampi the older participants had higher retrieval activation than the younger or middle-aged participants, and that this higher activation further correlated with decreased performance. Volumetric changes were not assessed.

While behavioural and volumetric studies indicated that the first changes in hippocampal structure and associated discrimination abilities can be detected in midlife, the associations between subfield volume, subfield activation and discrimination abilities across the lifespan remain unclear. In this study, we aimed to understand whether the relationships between discrimination performance (an index of pattern separation), hippocampal subfield volume and activity are age-dependent in a lifespan cohort (20–75 years). Given the non-linear age-associations with cognition^18^ and the suggested non-linear associations in hippocampal subfield activity during discrimination performance, we examined linear and non-linear models. We acquired ultra-high-field structural and functional MRI at 7 Tesla to accurately identify the hippocampal subfields using different modalities (functional and structural).

## Results

**Figure 1:**
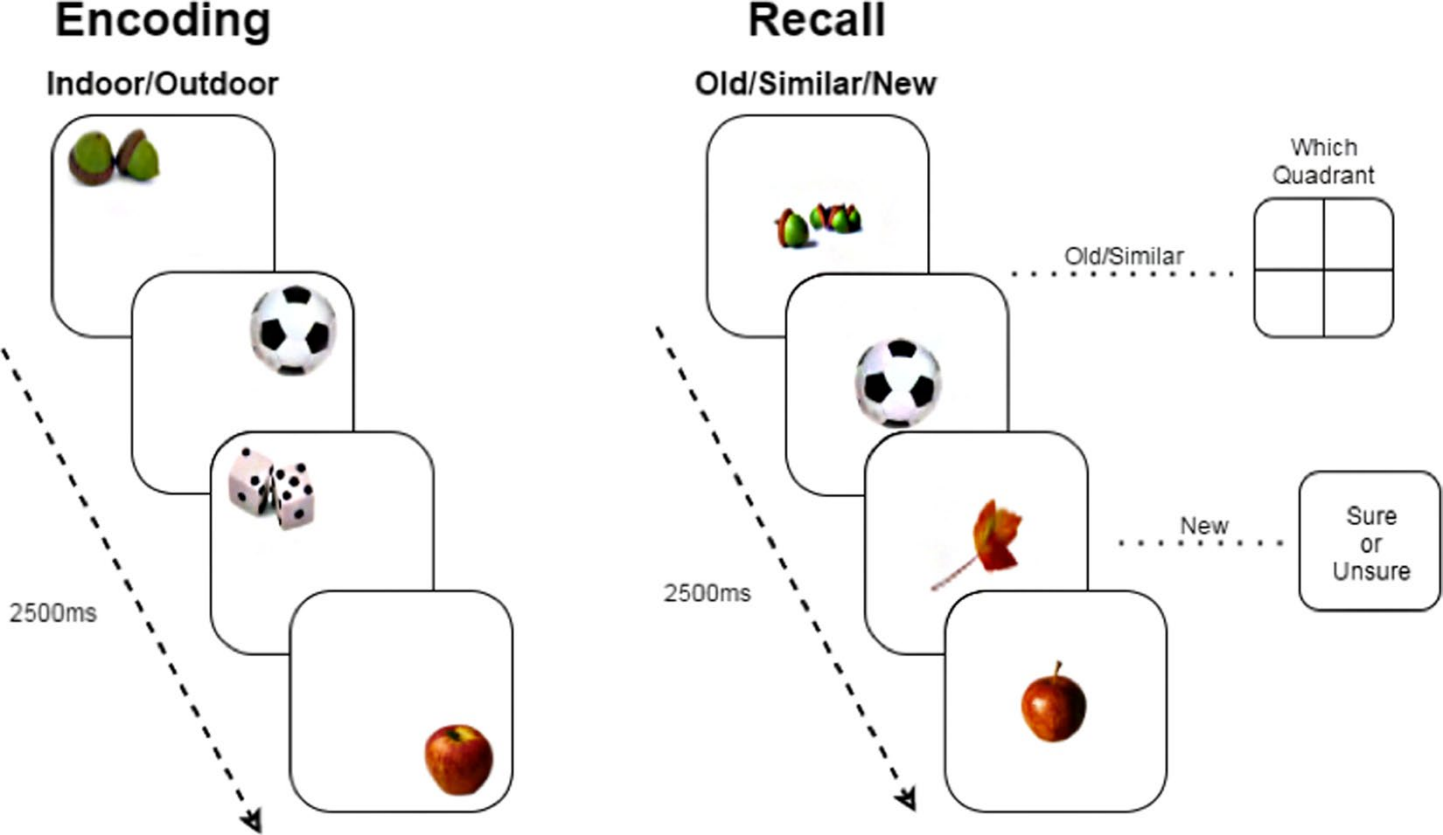
Experimental lure discrimination task. Note: Outline of lure discrimination task. During encoding, a daily life object is presented for 2500 ms and participants judge the nature of an object (“indoor” vs. “outdoor”). During retrieval, each object is again shown for 2500 ms. Participants first judge whether the object is “old” for targets, “similar” for lures, or “new” for foils. Next, for targets and lures, participants indicate in which quadrant the (original) was shown during encoding. For foils, participants judge how confident they are with their decision (“sure” vs. “unsure”). All images as well as the task are open access and freely available via (https://faculty.sites.uci.edu/starklab/mnemonic-similarity-task-mst/).

**Figure 2:**
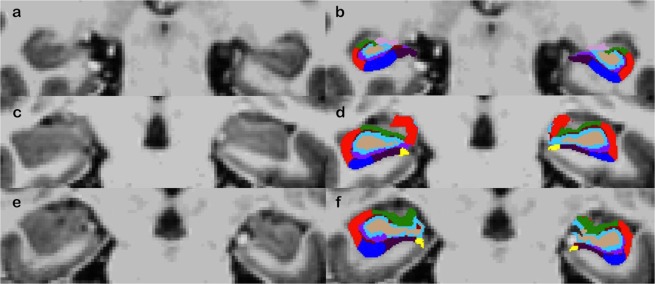
Hippocampal subfields segmentation. Note: Hippocampal subfield segmentation of three participants without (**a,c,e**) and with (**b,d,f**) superimposed the hippocampal subfield mask overlaid in coronal view. The first row (**a,b**) shows a young participant, the second row (**c,d**) shows a middle-aged participant, the old row (**e,f**) shows an older participant. Colours: CA1 (red), CA3 (green) and DG (blue).

Fifty-three participants were included in this observational study (30 female (56.6%), Age: *mean* = 45.05, *SD* = 18.2, *range* = 21–73)), who all underwent neuropsychological testing, structural and functional imaging at 7 T, during which the well-established lure discrimination paradigm was performed (Fig. 1). All included participants had neuropsychological test results within normal range and scored below the cut-off on the HDRS (<17) and MMSE (>24). A detailed overview of the sample characteristics and neuropsychological test results is provided in Table 1. FreeSurfer 6.0 was used to segment the hippocampal subfields (Fig. 2) and FSL to perform pre-processing of the functional data and compare functional activation between successful lure discrimination and unsuccessful lure discrimination.

**Table 1:** Demographics of sample.

N = 53	Mean (sd) n (%)	Range
***Demographics***		
Age (in years)	45.05 (18.2)	21–73
Female (%)	31 (57.4)	
Education^a^	5.51 (1.6)	2–8
***Neuropsychological assessment***		
HDRS-score	1.16 (1.8)	0–8
MMSE-score	29.38 (0.7)	27–30
WLT learning (number of words)	52.09 (8.3)	32–71
WLT delayed recall (number of words)	11.05 (2.8)	1–15
SWCT interference score	35.96 (15.6)	−2.3–69.8
LDST (number correct)	57.12 (10.6)	37–87
CST mental flexibility score	7.31 (4.7)	−2.1–19.8
Verbal Fluency animals (number words)	26.57 (6.2)	11–38
Verbal Fluency professions (number words)	18.41 (4.8)	11–32
Verbal Fluency M (number words)	17.20 (5.6)	7–31
LDI-score	0.16 (0.18)	−0.15–0.47
CRS	0.58 (0.25)	−0.02–0.90
***Subfield volume***^*b*^		
Left CA1	30.63 (3.2)	22.2–38.1
Right CA1	31.19 (3.3)	24.1–38.9
Left CA3	9.69 (1.1)	7.3–12.1
Right CA3	10.48 (1.2)	7.7–12.8
Left DG	13.88 (1.3)	11.2–16.2
Right DG	14.29 (1.4)	10.4–16.8
***Subfield activation***^*c*^		
Left CA1	3.09 (21.5)	−38.6–117.0
Right CA1	1.98 (17.1)	−52.7–47.1
Left CA3	−0.81 (29.1)	−92.1–65.1
Right CA3	2.31 (27.1)	−48.2–79.7
Left DG	8.11 (47.4)	−91.2–275.6
Right DG	5.51 (34.3)	−57.8–149.5

Note: HDRS: Hamilton Depression Rating Scale; MMSE: Mini Mental State Examination; WLT: Word learning test; SWCT: Stroop word colour test; LDST: Letter digit substitution test; CST: Concept shifting test; LDI: Lure Discrimination Index; CRS: Corrected Recognition Score. MMSE, WLT, Stroop, LDST, CST, and Verbal Fluency are corrected for age, sex, and education, CA: Cornu Ammonis, DG: dentate gyrus.

^ª^A standardized 8-point scale (CBS, 2011) was used to indicate educational level (range 1 = primary school to 8 = university).

^b^Subfield volume is expressed as raw volume in mm^3^ divided by intracranial volume in mm^3^ and multiplied by 1000 (arbitrary unit).

^c^Activation contrast is successful vs. unsuccessful pattern separation.

### Age associations with subfield volumes, subfield activity or LDI

**Figure 3:**
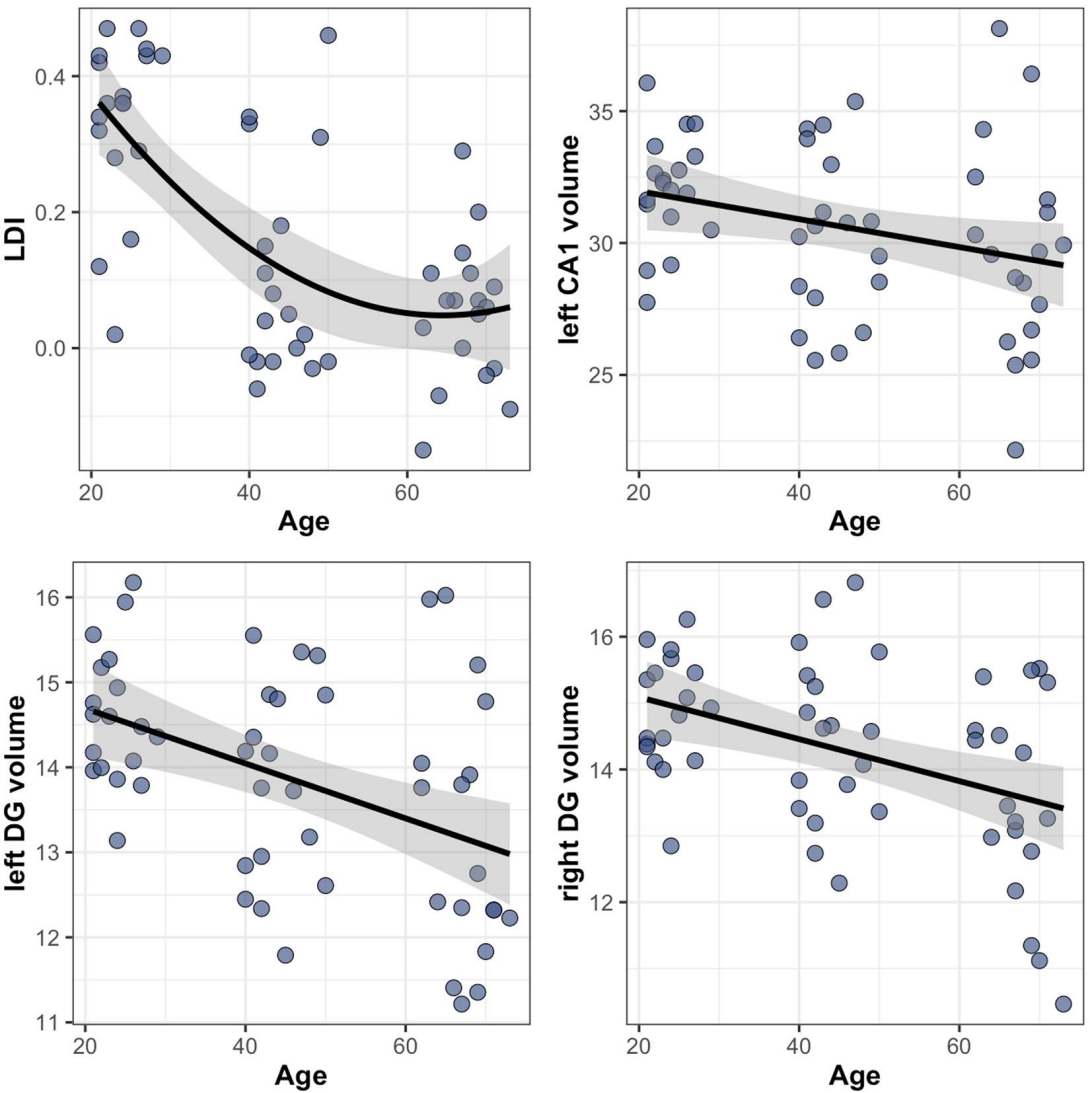
Relationships between hippocampal subfield volumes and discrimination performance. Note: Scatterplots showing the relationship between age and LDI (top left), age and left CA1 volume (top right), age and left DG volume (bottom left), and age and right DG volume (bottom right). The relationship between age and LDI is non-linear. Relationships between the three subfield volumes and age are linear. Only significant relationships are shown. Subfield volumes are is expressed as raw volume in mm^3^ divided by intracranial volume and multiplied by 1000 (arbitrary unit).

First, we aimed to investigate the association between age and subfield volumes, subfield activity, and lure discrimination performance (LDI). Given the nonlinear relationships between age and LDI, generalized additive models (GAM) were chosen to describe the relationship, allowing for a more data-driven approach to model smooth functions, which are more flexible than polynomial regressions. Comparisons of linear and nonlinear models revealed that smooth terms best described the association between age and LDI (*edf* = 7.00, *F* = 7.66, *p* < 0.001). Consistent with the literature, we observed linear negative age-associations between age and subfield volumes in the left CA1 (*ß*=−0.06, *t* = 2.32, *p* = 0.024), and the left and right DG (left: *ß*=−0.02, *t* = 3.05, *p* = 0.036, right: *ß*=−0.03, *t* = 2.52, *p* = 0.030) (see Fig. 3). In these models the linear term was not significantly different from the smooth age term, and hence the most parsimonious model was chosen. No linear or non-linear associations between age and subfield activation during pattern separation were found. Older age was linearly associated with lower Corrected Recognition Score (CRS), a measure of target recognition (*ß*=−0.007, *t* = −3.71, *p* < 0.001, see Supplementary Fig. S1).

### Age-varying associations between subfield volume and LDI performance

**Figure 4:**
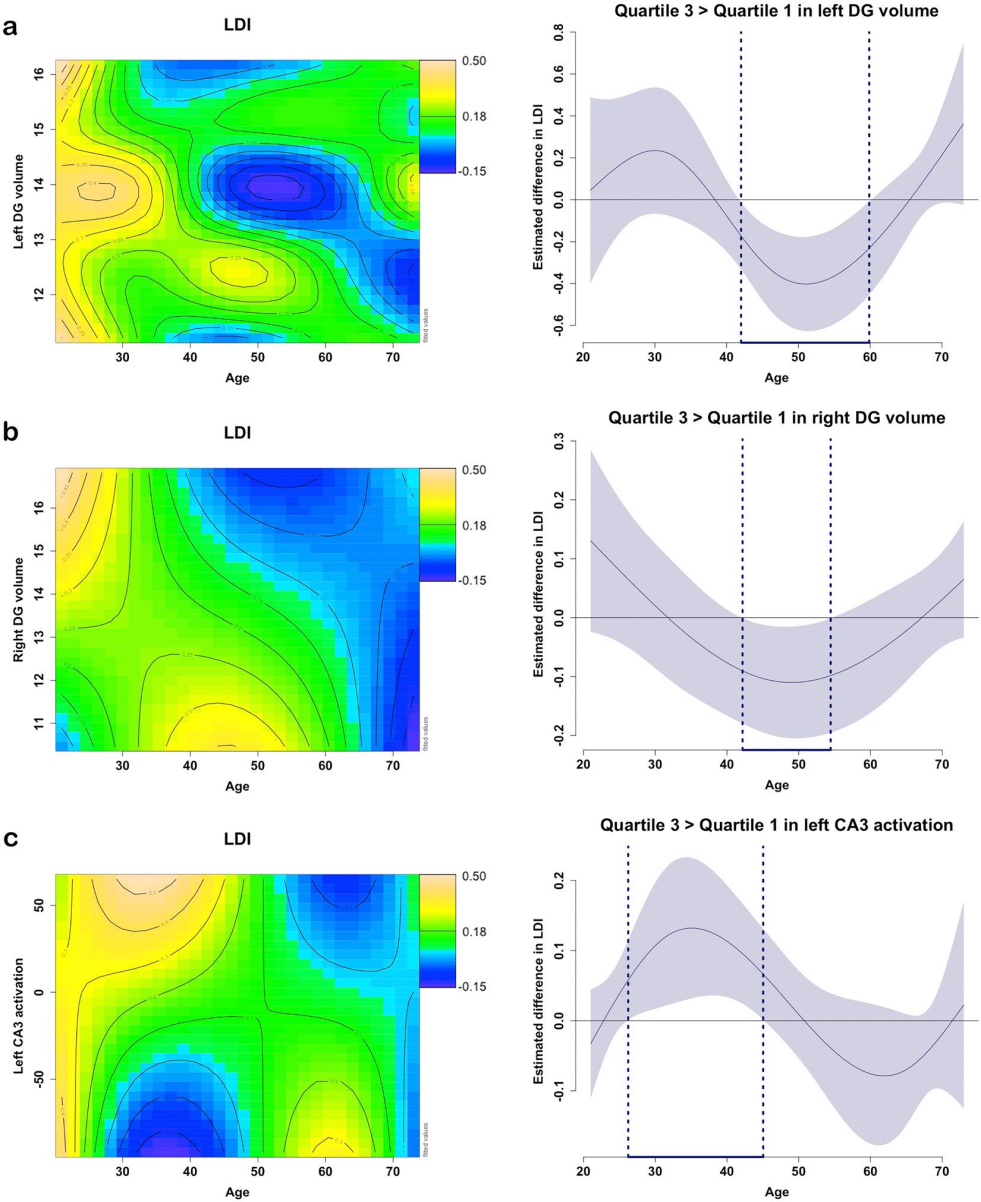
Non-linear relationships of age with hippocampal subfield volumes or activation predicting LDI-performance. Note: Age-varying associations between left (**a**) or right DG (**b**) volume with LDI performance; and age-varying associations between left CA3 pattern separation activation and LDI performance (**c**). The colour in the contour line codes the performance on the task (predicted values), with orange-yellow indicating a higher predicted score, green moderate, and blue poor scores. The distance between the iso-value (contour) lines indicates the steepness of the 3D plane. On the right side of each contour plot is a difference plot indicating during which age range a difference between higher and lower volume or activation (quartile 3 versus quartile 1) is associated with LDI performance (using floodlight analyses for curved associations).

Next, we examined whether the relationship between subfield volume and LDI varies with age. GAM tensor products were used to implement interactive effects. Model fit comparisons revealed that smooth modelling of the interaction “age by subfield volume” on LDI described the data best for the left and right DG. These 3-way non-linear interactions are visualized in the contour (or topographic) plots in Fig. 4A,B (Supplemental Table S1), where the colour scale indicates the LDI score and the distance between the contour lines indicates the steepness of the slope. For younger individuals (up to age 30), there is a vertical band of the yellow colour, indicating that they score relatively high on the LDI independent of the left DG volume (Y-axis). Similar relationships can be observed for the right DG volume (mixture of left and green values indicating moderate to good performance on LDI independent of the volume). We then estimated the age range at which a significant difference between individuals with high and low left DG volume on LDI performance can be detected (alpha <0.05) between 42.01 and 59.87 years. For the right DG volume, this age range was estimated at 42.54 to 54.94 years of age. These differences indicate that individuals in this age range who exhibited relatively higher DG volume, perform worse on the LDI than those with lower DG volume. For older individuals, there was a positive relationship between left DG volume and LDI scores (green zone: higher volume is associated with moderate-good scores; blue zone: lower volume is associated with worse LDI scores). We note that the association between LDI scores and the contrast between the third and first quartile in left DG volume (the green versus blue zones) was not steep enough to be significantly different in these older individuals. For the right DG, for older individuals (>60 years) a blue vertical band can be observed, indicating that performance on the LDI was low irrespective of right DG volume. To better understand the findings – in particular the ones in the middle-aged individuals -, we will also examine age-varying relationships between hippocampal subfield volumes and activation across the entire lifespan. There were no significant interactions between age and subfield volume for the left or right CA1 and CA3 when predicting LDI performance.

### Age-varying association between subfield activation and LDI performance

We investigated whether age modulates the relationship between subfield activation and LDI using the modelling methods described above. Model fit comparisons revealed that smooth modelling of the interaction “age by subfield activation” on LDI described the data best for the left CA3. In the left CA3 individuals with relatively higher activation was associated with better performance (green-yellow colours) when between the age of 26.25 and 44.64 years. For older individuals, the reversed pattern could be detected: between the age of 60.39 and 67.22, lower activation was associated with better performance (bottom green-yellow colour) and higher activation was associated with worse performance (top blue colours), though the floodlight analyses indicated that this was significant at a one-sided alpha of 0.05 (Fig. 4C).

No significant interactions between subfield activation and age on LDI were observed for the right CA3, CA1 or DG. For the left and right CA1, we observed independent negative age-effects with LDI and positive associations between activation and LDI. There were no interactions between age and activation on LDI performance. Younger age and higher activation were both associated with better performance. Left or right DG subfield activation did not contribute to LDI performance (Table 2 and Supplemental Table S2).

**Table 2:** Age, subfield volume or activation predicting LDI-performance.

	Volume and Age associations with LDI				Activation and Age associations with LDI			
	Model 1		Model 2	Model 3	Model 1		Model 2	Model 3
	Volume	Age smooth	Volume smooth x Age smooth	Volume x Age smooth	Activation	Age smooth	Activation smooth x Age smooth	Activation x smooth Age
Left CA1	0.70	7.77***	1.28	1.71	2.47*	17.56***	0.35	0.16
Right CA1	0.33	7.35***	0.95	1.54	2.60*	19.43***	0.98	0.61
Left CA3	1.01	8.09***	1.13	1.24	0.09	7.06***	2.64*^∆^	2.73*
Right CA3	0.99	7.33***	2.58	1.97	1.74	8.26***	3.34**^∆^	1.17
Left DG	1.43	8.21***	2.07*^∆^	2.34	1.20	8.11***	1.75	1.55
Right DG	0.34	7.34***	3.32*^∆^	1.58	1.77	16.81***	0.84	0.46

Note: Reported values are F-values. Covariates included in all generalized additive models are sex and education. Age is added as smooth variable in all models. Volume and activation are explored as smooth and linear terms. In models 1 the main effects (without the interaction in the model) are reported, for models 2 and 3 only the interactions are reported. More detailed statistical information is provided in Supplementary Tables 1 and 2. *Indicates significance (* p < 0.05; **p < 0.01; ***p < 0.001). ∆ indicates best model fit.

### Subfield volume association with activation

**Figure 5:**
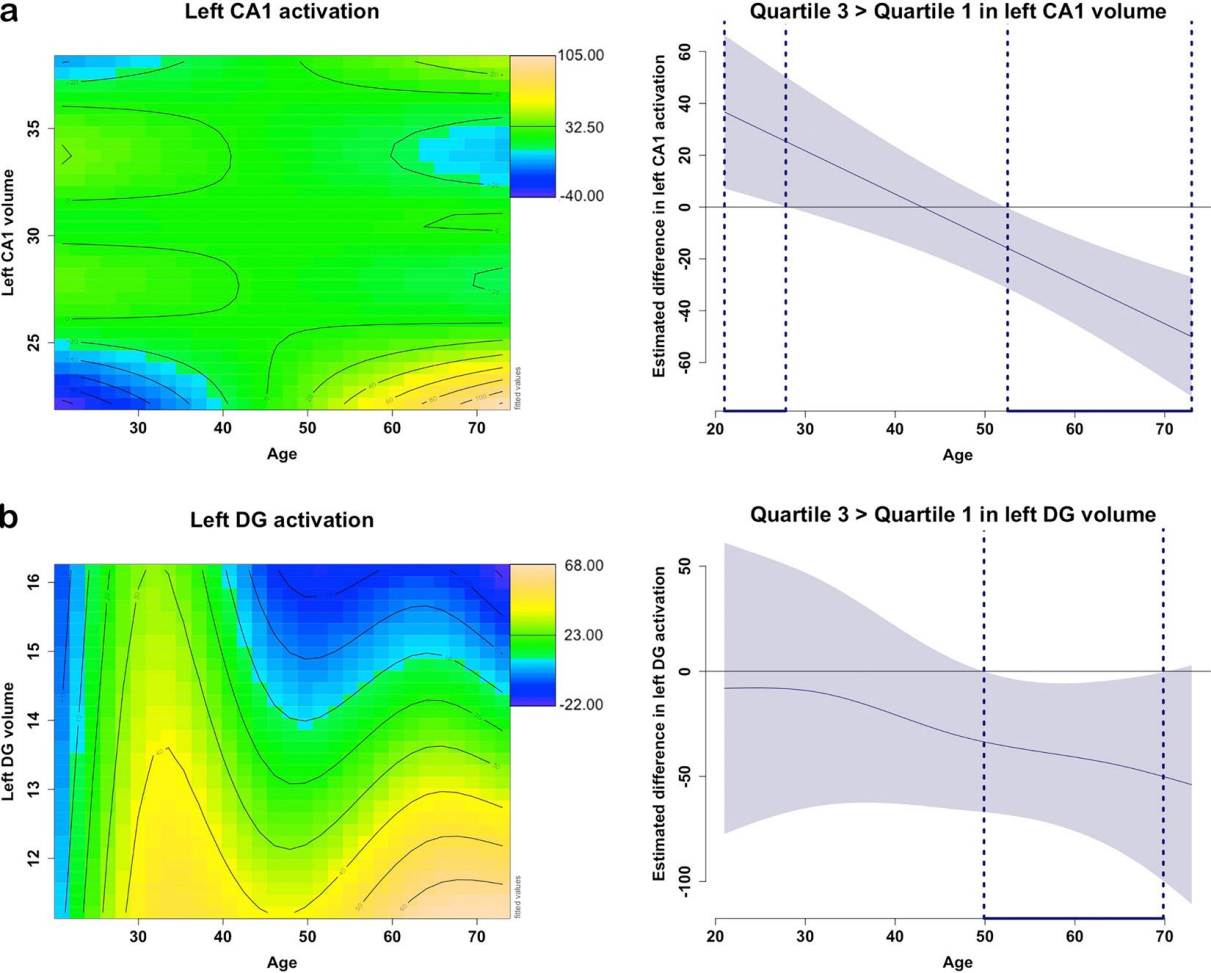
Non-linear relationships of age with hippocampal subfield volumes and activation. Note: Age-varying associations between left CA1 activation (**a**) or left DG activation (**b**) volume their respective volume. The colour in the contour line codes the performance on the task (predicted values), with orange-yellow indicating a higher predicted activation, green moderate, and blue lower activation. The distance between the iso-value (contour) lines indicates the steepness of the 3D plane. On the right side of each contour plot is a difference plot indicating during which age range a difference between higher and lower volume (quartile 3 versus quartile 1) is associated with activation (using floodlight analyses for curved associations).

In a next step we used GAM methods to examine whether subfield volume and subfield activation varied with age. Smooth interactions between subfield volume and age on subfield activity were found for the left CA1 and left DG (Fig. 5A,B). For the left CA1, we found a linear negative age-association interacting with a cubic CA1 volume to predict CA1 activation. As shown in Fig. 5A,B, this nonlinear association reveals that for individuals between the age of 21.00 and 28.35 years, greater left CA1 volume is associated with greater CA1 activation. (green region compared to the left blue bottom corner), while between the age of 55.67 and 73.00 years, greater left CA1 volume is associated with lower CA1 activation (bottom yellow corner contrasted with the blue blob). For the left DG, we note that for individuals between 48.84 and 73.00 years, higher left DG volumes were associated with lower left DG activation. For individuals younger than 50 years, left DG activation is relatively high irrespective of its volume (green-yellow zone). This is in agreement with our observation that in older individuals, lower activation and higher volume are associated with better LDI performance. The negative association between volume and LDI in middle-aged individuals may be related to an underlying pattern of higher neural activation. In the remaining subfields (the left and right CA1 and CA3, respectively) no significant interactive or independent age-varying associations between activation and volume were detected.

### LDI association with subfield volume and subfield activation

Finally, we investigated whether subfield volume and subfield activation were synergistically associated with LDI, independent of age. In all models age was added as a smooth covariate, and sex and education as linear covariates. For the left and right CA1, the interaction between smooth activation and linear volume was significant, but model inspection revealed that the edf was equal to 1. We therefore interpreted the more parsimonious linear models. We observed no significant linear interactions between subfield volume and subfield activation on LDI performance (Supplemental Table S3).

### Power calculation

We conducted a power calculation for the volumetric part of our analyses. To reach 80% power with an alpha-error probability of 0.05 and an estimated effect size of R^2^ = 0.221, as reported for the combined sample of young and old participants from a previous, similar study^16^, the calculated total sample-size was 38 participants, or 13 participants per age-group. The total sample-size of 53 participants in this study should thus be sufficient to detect a medium effect size. For 7 T fMRI-studies power calculations are less obvious. Our sample-size is comparable to previous work in the field at 3 T^11,13,16,19,20^.

## Discussion

The aim of this study was to investigate whether the relationship between hippocampal subfield volume or activity and discrimination performance varies across the lifespan, given that previous studies have indicated that around midlife the hippocampus is vulnerable to neurodegenerative processes. This volumetric change seems to occur simultaneously with lower discrimination performance, suggesting that 40–50 years of age is an important period in life to detect the first possible signs of age-related brain-behaviour changes and may also be a critical period to enhance brain resilience^24^. Our findings replicate previous studies reporting differences in discrimination performance between young and old participants^25^. We show that lure discrimination performance is negatively and non-linearly associated with increasing age. Our study adds important observations on the non-linear nature of this age-association during the lifespan, and its interplay with pattern separation-specific subfield volumes and activation patterns.

Interestingly, for the left and right DG we found that in middle-aged individuals (~40–50 years) lower hippocampal subfield volume was associated with higher discrimination performance. This age-group, between 40–50 years, is an important age group of interest, as many molecular and neuronal changes are manifested, ranging from hippocampal atrophy to increased amyloid-beta and tau protein accumulation^26–28^, that may set the stage for future cognitive decline. In fact, previous work has indicated that verbal episodic memory starts to decline around middle-age^29^. Understanding the neuronal correlates underlying subtle age-related cognitive changes and deciphering typical from atypical age-related decline will be crucial to improve early detection of persons at-risk for cognitive decline. As for the younger and older individuals a larger DG volume was associated with better LDI performance, consistent with findings from previous studies^16,30,31^.

In addition, we found that in the left CA3, higher activation is only associated with better performance in younger adults until about 45 years of age. The CA3 areas are crucially involved in pattern separation, and similar relationships between activation and LDI-performance in younger adults have been reported in the literature^9,32,33^. In contrast, in late adulthood (~60–70 years) lower activation in the left CA3 was at-trend level associated with better performance. Hyperactivation in the CA3 in older individuals has been related to worse performance in previous work^19^. This hyperactivity has been associated with reduced inhibitory activity by the recurrent auto-associative fibres in the CA3. These events, compounded by volume loss in the DG and lower performant pathway input, are assumed to lead to a pattern-completion bias^10^. The meaning of hyperactivation, whether it reflects compensatory or aberrant processes, has been a long debate^34^. The relationship with lower performance in our sample suggests an aberrant process, and we speculate that this could stem from neuronal network breakdown, potentially resulting from amyloid-induced hyperexcitability^35^. It is important to note that studies reporting compensatory mechanisms often find hyperactivation in regions outside the medial temporal lobe^36–38^. Hence, compensatory and aberrant processes may be co-existing but acting in different regions.

Activation during pattern separation in the left and right CA1 showed a negative relationship with LDI-performance, independent of age. The CA1 is a region typically, but not exclusively, recruited during pattern completion processes^32,39^ and only deteriorates with age with increasing difficulty of presented items^40^. A hypothetical explanation for why activation in this region contributes to predicting LDI-performance lies in the nature of the behavioural task. During retrieval, the similarity of the presented lures is manipulated, resulting in some lures being more or less similar to the object presented during encoding. For the more dissimilar lures, the CA1 is more strongly engaged, while for more similar lures, the CA3 and DG are more strongly engaged^9^.

While a coupling between age and subfield volumes for explaining subfield activation could have shed light on the interplay between structure and function of the subfields, we failed to find synergistic effects of subfield volumes and activation on task performance. However, we found that age modulated the association between subfield activation and volume in the left CA1 and DG. Interestingly, in the left CA1, from late midlife (>55 years), lower volume was associated with higher activation, which potentially hints at a hyperactivation of this subfield. Our data does not provide conclusive evidence to determine whether this activation is related to aberrant neural processes or underlying pathology.

## Limitations

This version of the discrimination paradigm had a high difficulty level, as it included an additional active location-retrieval component, while the classic task focuses solely on discrimination and recognition^15^. While it is important to prevent ceiling effects in younger individuals, a more difficult task may demotivate older individuals. Our participants did not report lack of motivation after testing. As indicated by the CRS, our participants showed a normal distribution of scores on target recognition, excluding possible floor effects due to task difficulty. Nonetheless, it may be interesting to explore the use of adaptive discrimination tasks that titrate difficulty level on a subject-by-subject level. We are also aware that by recruiting individuals within three pre-defined age groups, we are missing out data and interpolating our effects in between these age groups, but based on previous findings^41^ we do not expect strong brain-behaviour changes in the 10-year time period in between the successive age groups.

## Conclusion

In conclusion, we showed age-dependent, non-linear associations in lure discrimination performance. For older individuals, lower DG volume and higher CA3 activation was associated with worse LDI performance, suggesting that this higher activation may be an indication of aberrant neurodegenerative-related processes. In fact, higher activation in the CA1 and DG was associated with lower volumes in these subfields. Importantly, we observed specific brain-cognition patterns during midlife. For individuals around 40–50 years old, we observed that greater left and right DG volume, but greater activity in the CA3 was associated with lower LDI performance. These findings highlight that midlife is a critical time in life, where subtle brain changes related to memory decline can be observed. These findings reiterate the current opinion that starting interventions during midlife to delay or modify cognitive decline and potentially neurodegenerative disorders is of utmost importance^24,42^.

## Methods

### Participants

Fifty-three individuals (30 female (56.60%), Age: *mean* = 45.05, *SD* = 18.2, *range* = 21–73) were recruited from the general population via advertisements. Participants were stratified into three age groups (20–30 years old (n = 18), 40–50 years old (n = 18) and 60–80 years old (n = 17). The choice of these age groups was based on literature indicating that cerebral changes are most pronounced in these phases of life^41^. In addition, studies in the field of cognitive neuroscience have suggested that memory performance stays relatively stable until midlife and then declines with age^43^. Thus, this sampling choice should provide a reasonable representation of the population and brain-behavior differences of interest, while allowing a sample-size that is manageable given the complexity of 7 T MR-scanning. Inclusion was allowed if participants were right-handed and had normal or corrected-to-normal vision and absence of contra-indications for MR-scanning, major neurological disorders, depression, and hypertension (with or without treatment).

Informed consent was obtained from each participant prior to participation. Each participant underwent a comprehensive neuropsychological assessment included the Mini Mental State Examination (MMSE,^44^), the Dutch version of the 15-word learning task^45^ for episodic memory (learning and delayed recall), the concept-shifting test^46^ and the Stroop-Colour Word-test for attention and executive functioning^47^, letter substitution test for processing speed^48^, and a category and letter verbal-fluency task for language^49^. Performance below 2 standard deviations (SD) from the mean, corrected for age, sex, and education in these domains led to exclusion from the study. Depression was assessed with the Hamilton Depression Rating Scale (HDRS,^50^). Participants with scores above 17 were excluded from analysis^51^, as depression can influence memory performance. In total, seven individuals (out of n = 60) were excluded prior to participation in the fMRI study and are not included in the current analyses. Written informed consent was obtained from each individual. The study was approved by the local ethics committee of the Faculty of Psychology and Neuroscience at Maastricht University and was carried out in accordance with the Helsinki guidelines.

### Experimental pattern-separation task

During fMRI-scanning all participants performed an object version of a validated robust mnemonic object discrimination task^10,15^ with a location retrieval component^52^. The location retrieval component is intended to assess source memory but since it is not a direct assessment of the discrimination process, it was not included in the current analysis. Reliability measures for this task are not available. During encoding participants saw 150 every-day objects, appearing in one of the four corners of the screen, and were instructed to make an “indoor-outdoor” judgement. The judgement was added to ensure adequate attention and processing of the object. Stimulus presentation for each object was 2500 ms. During retrieval participants were presented with targets (n = 50), similar lures (n = 100) and novel foils (n = 50). Stimulus presentation was the same as during encoding (2500 ms). All objects were presented at the centre of the screen during retrieval. Targets were objects that were present during both encoding and retrieval, while foils were new objects that were absent during encoding. Similar lures were objects that resembled those presented during encoding, but differed along one or more dimensions (angle of presentation, brand, colour, etc.). Those critical lure trials allowed for an assessment of discrimination performance, which is sensitive to the integrity of the pattern separation process. Participants were asked to make two judgements, the first one regarding memory for the object (“Old”, “Similar” or “New”). Old and Similar judgments were followed by a second judgement made with regard to the quadrant in which the object was presented during encoding. New judgments were followed by a second judgment that was a confidence judgment (“Sure vs. “Unsure”).

Discrimination performance was measured using the Lure Discrimination Index (LDI), which controls for response bias and is calculated as p(“Similar”|Lure) – p(“Similar”|Foil). Probabilities were calculated by dividing the number of responses by the total number of responses to lures (e.g., p(“Similar”|Lure) is calculated by dividing number of correct lure trials called “Similar” by total number of lure trials). Memory for targets was also similarly calculated using a Corrected Recognition Score (CRS) operationalized as p(“Old”|Target) – p(“Old”|Foil) and is reported here for the sake of completion. Trials without a behavioural response were not taken into account for the calculation of the LDI or CRS.

### MR-scanning

FMRI data were acquired on a MAGNETOM 7 T whole-body magnet scanner (Siemens Healthineers, Erlangen, Germany) with a 32-channel head coil (Nova Medical, Wilmington, MA, USA) during both encoding and retrieval in anterior-posterior direction, covering the whole brain with the following parameters: voxel size = 1.25 mm isotropic (90 slices), FoV = 200 mm, TR = 1400 ms, TE = 19 ms, Flip Angle = 70°, interleaved acquisition, and a multi-band GRAPPA acceleration factor of 3 (acquisition time = 16 min 30 s minutes for retrieval). Reversed phase-encoding blips were collected, resulting in pairs of images with distortions in opposite directions. Furthermore, a high-resolution anatomical Magnetization Prepared 2 Rapid Acquisition Gradient Echoes (MP2RAGE) image^53^ was acquired in 240 slices with 0.7 mm isotropic voxel size, covering the whole brain (FoV = 224 mm, TR = 5000 ms, TE = 2.47 ms, Flip Angle = 5°/3°, Inversion time=900/2750 ms and acquisition time = 9 min 42 s.). Between encoding and retrieval there was a resting period of 6 minutes, each representing a different run.

### Hippocampal subfields

Hippocampal subfields were automatically segmented from the T1-weighted images with FreeSurfer version 6.0, using the built-in automated reconstruction process^54,55^. The technical details of these procedures are described in prior publications^55^. Briefly, the processing involves intensity normalization of the T1-weighted images, skull-stripping, segregating left and right hemispheres, removing brainstem and cerebellum, correcting topology defects, defining the borders of grey and white matter, and of grey matter and cerebrospinal fluid (CSF), and parcellating cortical and subcortical areas. Further, using FS’s native visualization toolbox and in-house tools we visually inspected and, if necessary, edited each image for over- or under-estimation of the grey/white matter boundaries and to identify brain areas erroneously excluded during skull stripping. In addition, we checked if the hippocampal subregion mask was well positioned. The current version of FreeSurfer uses a Bayesian inference approach combined with a novel atlas algorithm of the hippocampal formations, based on an ultra-high resolution (0.13 mm isotropic) *ex vivo* MRI atlas created from 15 postmortem brains. In addition, the approach was validated in an independent *in vivo* 1 mm MRI resolution data-set of 39 individuals, and was shown to be superior to its predecessor (FreeSurfer v.5.0.3) that was based on *in-vivo* data only. FreeSurfer v6.0 further has a high reliability (intraclass correlation coefficient = 0.9 or higher for subfields CA1, CA3, DG, subiculum, and other), which is also superior to the previous version of Freesurfer^56^. Identification of hippocampal subfields using FreeSurfer 6.0 should be considered probabilistic, as it uses prior knowledge from *ex vivo* brains scanned at 7 T MRI combined with the available contrast information from MR images (Iglesias, et al., 2015). Moreover, combining the contrast of a T2- with T1-weighted image may improve FreeSurfer’s segmentation, however, high-resolution T2-weighted images were not available in the current study.

Hippocampal subfields considered in the analyses are CA1, CA3, and dentate gyrus (DG) (see Fig. 4). Subfield volume was expressed in arbitrary units as actual volume divided by intracranial volume, multiplied by 1000, consistent with other studies. Left and right hemispheres were investigated separately.

### fMRI-analysis

FMRI preprocessing was completed with FSL version 5.0.9 (Woolrich *et al*., 2009). First, the functional images were motion corrected with respect to the last image with a 6-dof transform using MCFLIRT^57^. The motion outliers were determined as the frames with framewise-displacement higher than 0.9 (Power *et al*., 2012). These motion outliers were later added to the general linear model (GLM) to be regressed out. To reduce EPI-distortions, which are greater at 7 T than at 3 T, the susceptibility-induced off-resonance field was estimated and applied with FSL-TOPUP by employing the last 5 frames of each fMRI scan and their closest time-wise reversed phase encoding blips^58^. To further remove motion artifacts, an independent component analysis (ICA-AROMA) was applied^59^. Finally, the data were smoothed with a 1.5mm^3^ Gaussian kernel, which is roughly the size of one voxel, allowing us to investigate our tiny regions of interest, without losing information due to smoothing. To register the BOLD fMRI images to the T1, a boundary-based registration was applied using FSL’s epi_reg on the white and gray matter segmentations from FreeSurfer. The hippocampal subfields extracted from FreeSurfer, as well as CSF and white matter masks, were projected to the native fMRI space by means of the inverse of this transform. This ensured that the subfield segmentation had the same spatial resolution as the fMRI data. A GLM was implemented to compare the BOLD-response during correct lure discrimination and incorrect lure discrimination across groups. The average time course of CSF and white matter, as defined by FreeSurfer, were extracted for inclusion as confound regressor in the GLM model to minimize partial volume effects. In addition, the motion outliers and the 6 first order motion parameters were also added to the model. The behavioral regressors were modeled as boxcar functions with a variable epoch model. A double gamma and a temporal derivative were used as basis functions to be convolved with the GLM fitting. Functional activation in the hippocampal subfields of interest was extracted with featquery, which included weighing for the interpolation values of the downsampling of the hippocampal subfield masks and masks were subsequently thresholded at value 0.5 to mitigate possible partial volume effects. Activation was extracted for the contrast successful lure discrimination> unsuccessful lure discrimination (emphasizing pattern separation). A successful lure discrimination is defined as a lure that was identified as being similar, whereas an unsuccessful lure discrimination was a lure that was identified as old. A response of “old” to a lure item would suggest that the participant was more biased towards pattern completion, whereas an accurate response of “similar” to a lure would suggest a bias towards pattern separation instead. Lures subsequently identified as new were modelled to serve as an arbitrary baseline for the other conditions, but was not considered in the analysis.

### Statistical analyses

Statistical analyses were performed with R software version 3.5 (www.r-project.org). Demographics are provided using the means and standard deviations. General additive models were carried out with the mgcv package. GAM is a type of modelling that is more data-driven than standard regression modelling^60^. GAM is able to fit any form of relationship, linear or non-linear, between variables without explicit assumptions on the shape of the modelled fit and with more flexibility than polynomial forms. Where polynomials model global expansions (data of the 20-year old individual contribute to the fit of the 80-year old), GAM allows for local expansions, resulting in a better fit. We applied smooth regression splines in additive models and tensor product splines for interactive models.

First, we investigated the linear and non-linear age-associations with LDI, subfield volumes or subfield activations. Sex and education were added as covariates in all models. In a second step, we assessed age-varying relationships between LDI performance and subfield volume or activation. To this end, LDI was taken as outcome variable, subfield volume or activation as predictor variable in interaction with age. Sex and education were added as covariates to all models. All models were performed in the entire group. Finally, we investigated possible age-varying associations between subfield activation and subfield volume, with activation as outcome variable and subfield volume as predictor, and sex and education as covariates. We also investigated the interaction between subfield volume and subfield activation on LDI performance corrected for smooth age, education and sex. In all analyses smooth terms were compared with linear terms using the Akaike Information Criteria (AIC) and the Wald test on an analysis of deviance to guide model selection and help guard against over-fitting. For the GAM models with interactions, we determined the age-range where curve differences between the first and third quartile for the subfield predictor interacting with age were significant in predicting the outcome (floodlight analyses including the entire age range). Difference between the first (25–50%) and third (50–75%) quartile were chosen to detect the most salient associations. These quartiles were chosen to reflect the interquartile range of the data and to eliminate the influence of values at the endpoints of the distribution. The region of significance was identified by the 95% confidence interval that did not include zero. Statistical significance was set at p < 0.05.

## Supplementary information

Supplementary materials.

## Data Availability

Data used in the current study is available from the corresponding author on reasonable request and in accordance with the EU legislation on the general data protection regulation.
