# Supplementary material for: Associations between pattern separation and hippocampal subfield structure and function vary along the lifespan: A 7 T imaging study
Source: Sci Rep. 2020 May 5;10:7572. doi: 10.1038/s41598-020-64595-z (PMC7200747; doi:10.1038/s41598-020-64595-z)
Supplement: Supplementary file 1 — Supplementary materials. [file 41598_2020_64595_MOESM1_ESM.pdf]

Supplementary material

Associations between pattern separation and hippocampal subfield structure and function vary along the lifespan: A 7T imaging study

Authors:

Joost M Riphagen, MD, PhD

Lisa Schmiedek, PhD

Ed HBM Gronenschild, PhD

Michael A. Yassa, PhD

Nikos Priovoulos, PhD

Alexander T. Sack

Frans RJ Verhey, MD PhD

Heidi I.L. Jacobs, PhD

Supplementary Table 1: Overview of linear and generalized additive models of hippocampal subfield volume predicting LDI

	model	edf	F-value	p-value	AIC	Model comparison (p-value)
**Left CA1**	GAM with interaction of smooth age and linear volume	1.81	1.71	0.187	435.92	
	GAM with interaction of smooth age and smooth volume	2.76	1.28	0.337	434.21	0.125
**Right CA1**	GAM with interaction of smooth age and linear volume	1.84	1.54	0.214	437.57	
	GAM with interaction of smooth age and smooth volume	2.74	0.95	0.432	435.65	0.159
**Left CA3**	GAM with interaction of smooth age and linear volume	1.85	1.24	0.299	435.99	
	GAM with interaction of smooth age and smooth volume	3.98	1.13	0.332	434.37	0.117
**Right CA3**	GAM with interaction of smooth age and linear volume	1.91	1.97	0.139	432.86	
	GAM with interaction of smooth age and smooth volume	1.92	2.58	0.074	431.61	0.0236*
**Left DG**	GAM with interaction of smooth age and linear volume	2.01	2.34	0.097	433.89	
	GAM with interaction of smooth age and smooth volume$^{\Delta}$	10.41	2.07	0.042*	422.38	0.004*
**Right DG**	GAM with interaction of smooth age and linear volume	1.92	1.58	0.199	431.67	

	model	edf	F-value	p-value	AIC	Model comparison (p-value)
	GAM with interaction of smooth age and smooth volume$^{\Delta}$	2.03	3.32	0.030*	430.93	0.005*

Note: GAM: generalized additive model, edf: effective degrees of freedom, Covariates in all models are sex and education. Δ indicates best model fit based on statistical model comparisons.

Supplementary Table 2: Overview of linear and generalized additive models of hippocampal subfield activation predicting LDI

	model	edf	F-value	p-value	AIC	Model comparison (p-value)
**Left CA1**	GAM with linear activation and smooth age (no interaction) $^{\Delta}$	Age: 1.77<br>Activation: 0.21	17.56<br>2.47	<0.001*<br>0.017*	430.46	0.008*
	GAM with interaction of smooth age and linear activation	1.00	0.16	0.689	432.21	0.858
	GAM with interaction of smooth age and smooth activation	2.89	0.35	0.692	432.21	0.305
**Right CA1**	GAM with linear activation and smooth age (no interaction) $^{\Delta}$	Age: 1.88<br>Activation: 0.27	19.43<br>2.60	<0.001*<br>0.012*	428.45	0.019*
	GAM with interaction of smooth age and linear activation	1.53	0.61	0.439	427.72	0.307
	GAM with interaction of smooth age and smooth activation	3.07	0.98	0.425	427.54	0.188
**Left CA3**	GAM with linear activation and smooth age (no interaction)	Age: 7.02<br>Activation: 0.01	7.06<br>0.08	<0.001*<br>0.932	428.49	0.002*
	GAM with interaction of smooth age and linear activation	3.29	2.73	0.038*	430.91	0.069

	GAM with interaction of smooth age and smooth activation$^{\Delta}$	3.27	2.64	0.042*	431.08	0.031*
**Right CA3**	GAM with linear activation and smooth age (no interaction) $^{\Delta}$	7.12	8.26	<0.001*	424.74	<0.001*
	GAM with interaction of smooth age and linear activation	2.78	1.17	0.369	435.70	0.001*
	GAM with interaction of smooth age and smooth activation	14.78#	3.34	.002*	410.80	<0.001*
**Left DG**	GAM with linear activation and smooth age (no interaction) $^{\Delta}$	7.03	8.11	<0.001*	426.81	<0.002*
	GAM with interaction of smooth age and linear activation	1.67	1.55	0.229	432.92	0.008*
	GAM with interaction of smooth age and smooth activation	4.97	1.75	0.174	431.91	0.099
**Right DG**	GAM with linear activation and smooth age (no interaction) $^{\Delta}$	1.87	16.81	<0.001*	433.20	0.010*
	GAM with interaction of smooth age and linear activation	1.38	0.46	0.688	434.76	0.527
	GAM with interaction of smooth age and smooth activation	3.79	0.84	0.564	433.45	0.175

Note: GAM: generalized additive model, edf: effective degrees of freedom. Covariates in all models are sex and education. Δ indicates best model f based on statistical model comparisons. #Basis dimensions, model fit and residual check indicate overfitting.

Supplementary Table 3: Overview of linear and generalized additive models of subfield volume and activation predicting LDI

	model	edf	F-value	p-value	AIC	Model comparison (p-value)
**Left CA1**	Interaction of smooth activation smooth and smooth volume	1.00	3.24	0.078	424.73	
	Interaction of linear activation and smooth volume$^{\Delta}$	1.00	3.28	0.076	424.77	0.632
	Interaction of smooth activation and linear volume	1.00	6.13	0.016*	430.53	0.018*
	Interaction of linear activation and linear volume	0.01	0.37	0.712	433.99	0.786
**Right CA1**	Interaction of smooth activation smooth and smooth volume$^{\Delta}$	1.59	1.71	0.162	423.87	
	Interaction of linear activation and smooth volume	1.55	1.72	0.142	423.45	0.712
	Interaction of smooth activation and linear volume$^{\#}$	1.00	7.37	0.009**	428.79	0.031*
	Interaction of linear activation and linear volume	-0.04	0.85	0.395	431.27	0.506
**Left CA3**	Interaction of smooth activation smooth and smooth volume	1.73	1.56	0.243	425.05	
	Interaction of linear activation and smooth volume	1.69	1.34	0.251	425.23	0.498
	Interaction of smooth activation and linear volume	1.00	0.24	0.624	428.44	0.075
	Interaction of linear activation and linear volume	0.07	1.02	0.311	430.26	0.422
**Right CA3**	Interaction of smooth activation smooth and smooth volume	1.80	0.98	0.394	426.29	

	Interaction of linear activation and smooth volume	1.78	0.87	0.428	426.50	0.837
	Interaction of smooth activation and linear volume	1.00	2.82	0.099	424.91	0.091
	Interaction of linear activation and linear volume	0.05	0.66	0.511	425.79	0.289
**Left DG**	Interaction of smooth activation smooth and smooth volume$^{\Delta}$	7.03	8.11	0.063	421.25	
	Interaction of linear activation and smooth volume	1.00	2.70	0.105	425.95	0.033*
	Interaction of smooth activation and linear volume	1.53	0.84	0.326	426.31	0.022*
	Interaction of linear activation and linear volume	-0.02	1.03	0.311	428.64	0.633
**Right DG**	Interaction of smooth activation smooth and smooth volume	1.00	1.36	0.249	427.18	
	Interaction of linear activation and smooth volume	1.00	2.41	0.128	424.74	0.197
	Interaction of smooth activation and linear volume	1.42	0.73	0.344	426.61	0.163
	Interaction of linear activation and linear volume	-0.04	0.82	0.412	430.13	0.149

Note: edf: effective degrees of freedom. Covariates in all models are age, sex and education. Δ indicates best model fit based on statistical model comparisons. #Basis dimensions, model fit and residual check indicate overfitting

Supplementary Figure 1: Associations of Age with LDI, and Age with Corrected Recognition Score

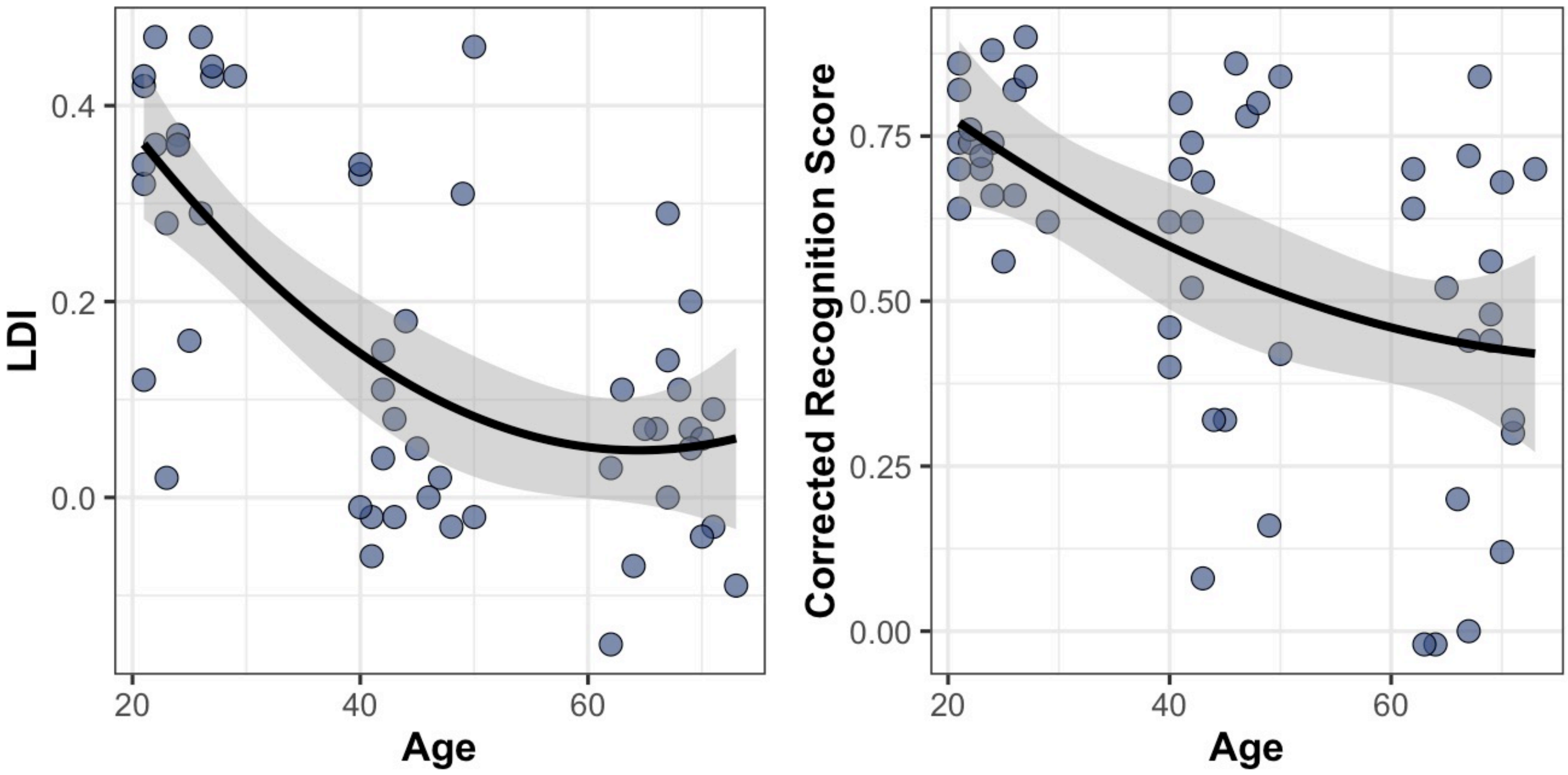

Note: Scatterplots showing the relationship between age and LDI (left) and age and Corrected Recognition Score (CRS) (right). The association between age and LDI is non-linear, the association between age and CRS is linear.
